# Selecting suitable chemotherapies for PD-1/PD-L1 blockade to optimize the tumor immune microenvironment

**DOI:** 10.18632/oncotarget.26028

**Published:** 2018-08-24

**Authors:** Shohei Koyama, Izumi Nagatomo, Takashi Kijima, Atsushi Kumanogoh

**Affiliations:** Department of Respiratory Medicine and Clinical Immunology, Graduate School of Medicine, Osaka University, Osaka, Japan; Department of Immunopathology, WPI Immunology Frontier Research Center (iFReC), Osaka University, Osaka, Japan

**Keywords:** cytotoxic chemotherapy, irinotecan, immune checkpoint inhibitor, PD-L1

Subsets of various malignancies exhibit clinical responses to immunotherapy with PD-1/PD-L1 blocking antibodies, so-called immune checkpoint inhibitors (ICIs). Currently, five anti–PD-1 and anti–PD-L1 antibodies have been approved by the US FDA as ICIs for eleven cancer indications (reviewed in [1]). In comparison with conventional cytotoxic chemotherapies, ICIs are superior for long-term disease control in sensitive patients. Multiple biomarkers, including PD-L1 expression in tumor cells and tumor mutation burden, are useful for predicting the therapeutic response to ICIs before treatment is initiated, and can discriminate between primarily sensitive tumors and resistant tumors [1]. In addition, investigations of patients whose tumors were initially sensitive but ultimately developed adaptive or acquired resistance have revealed additional mechanisms involved in resistance to PD-1/PD-L1 blockade [2]. Numerous studies, including preclinical models, have shown that tumors have multiple immune-evasion mechanisms, and that spatio-temporal genetic and immunological heterogeneities develop during tumor progression under the selective pressure of treatment [3, 4]. Therefore, to overcome resistance to ICI treatment, it is critical to co-target the immune checkpoint molecules with these additional immune-evasion mechanisms.

Recent clinical trials demonstrated the benefit of combination chemotherapies with PD-1/PD-L1 blocking antibodies in non–small cell lung cancer [5]. However, several questions persist regarding the administration of cytotoxic therapies in conjunction with these ICIs; for instance, what types, intensity, or schedules of cytotoxic reagents are most suitable for combination therapy with ICIs? The answers to these questions may vary with the tissue origin of the malignancy, patient age, and number of prior regimens. Mechanisms of chemotherapy-induced immune modulation have been extensively investigated in preclinical murine models, in which dose- and time-dependent immune responses can be evaluated in multiple tissues; however, syngeneic mouse models do not completely reproduce the tumor immune microenvironment in humans [6]. Certain types of cytotoxic reagents, such as oxaliplatin [7] and gemcitabine [8], can enhance the efficacy of PD-1/PD-L1 blockade; however, only a few immunogenic chemotherapies are reported to be effective in the context of combination treatment with PD-1/PD-L1 blocking antibodies.

In this issue, Iwai *et al.* [9] report the potential immunogenic function of a topoisomerase I inhibitor, irinotecan, as a combination partner for anti–PD-L1 antibodies. Although irinotecan is commonly utilized for several types of tumors, including colorectal, gastric, lung, and breast cancers, few studies have investigated immune modulation by this reagent. The authors performed immune profiling of tumors, as well as peripheral blood and sentinel lymph nodes, at different time points after initiation of treatment in a murine breast cancer model.

They found that irinotecan exerted three major effects on the tumor immune microenvironment: 1) a cytotoxic effect on tumor cells; 2) modulation of the microenvironment via a reduction in the abundance of Foxp3+ regulatory T cells on days 4 and 8 after injection, and of myeloid-derived suppressor cells (MDSCs) at day 4, leading to elevated proliferation and IFNγ production by tumor-specific CD8 T cells; and 3) an increase in MHC class I and PD-L1 mediated by both direct effects on tumor cells and IFNγ in activated T cells (Figure 1). These immune-modulating functions of irinotecan resulted in a supra-additive effect when the drug was administered with anti–PD-L1 blocking antibodies. Although efficacy in human patients still needs to be confirmed by a clinical trial, accumulating profiles of the immunogenic effects of cytotoxic chemotherapies in preclinical models provides the rationale for choosing specific reagents for combination therapy with PD-1/PD-L1 blocking antibodies. Given that this compound is already being used for treatment of several cancers, it would be feasible to initiate a combination clinical trial with ICI.

**Figure 1:**
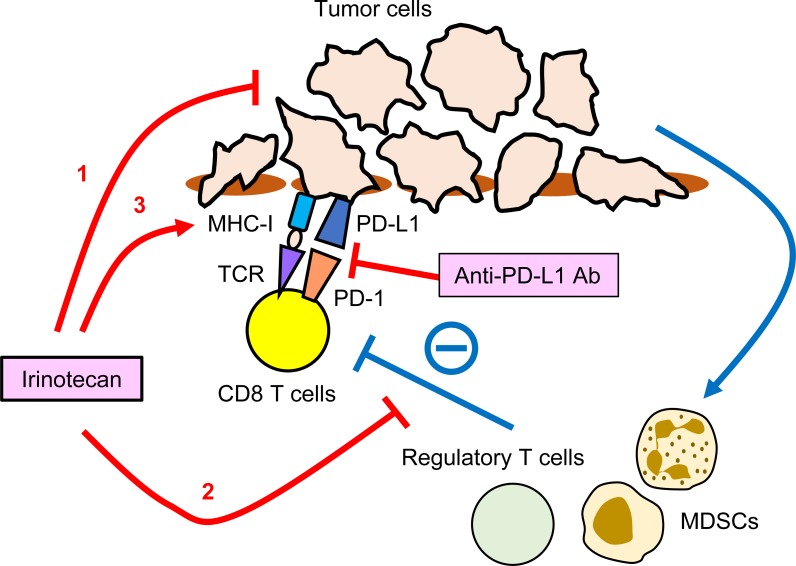
Potential targets of irinotecan in tumor immune-microenvironment **1.** A cytotoxic effect on tumor cells. **2.** A reduction in the abundance of Foxp3+ regulatory T cells and myeloid-derived suppressor cells (MDSCs), leading to elevated proliferation and IFNγ production by tumor-specific CD8 T cells. **3.** An increase in MHC class I and PD-L1 mediated by both direct effects on tumor cells and IFNγ in activated T cells.

## References

[1] Ribas A (2018). Science.

[2] Sharma P (2017). Cell.

[3] McGranahan N (2015). Cancer Cell.

[4] Chen DS (2017). Nature.

[5] Gandhi L (2018). N Engl J Med.

[6] Cook AM (2016). Curr Opin Immunol.

[7] Song W (2018). Nat Commun.

[8] Wang C (2018). Sci Transl Med.

[9] Iwai T (2018). Oncotarget.

